# Tobacco smoking history and antibiotic-resistant infection: a systematic review and meta-analysis

**DOI:** 10.3389/fpubh.2026.1935025

**Published:** 2026-10-07

**Authors:** Yusuff Adebayo Adebisi, Adeola Bamisaiye

**Affiliations:** 1 College of Social Sciences, University of Glasgow, Glasgow, United Kingdom; 2 Department of Clinical & Experimental Medicine, University of Catania, Catania, Italy; 3 Centre for Tropical Medicine and Global Health, Nuffield Department of Medicine, University of Oxford, Oxford, United Kingdom

**Keywords:** antibiotic resistance, antimicrobial resistance, drug-resistant tuberculosis, meta-analysis, smoking history, systematic review, tobacco smoking

## Abstract

**Background:**

Antibiotic resistance is a major global health threat, but host-level and behavioral contributors to resistant infection remain insufficiently quantified. Tobacco smoking impairs respiratory and systemic host defense and is a plausible, modifiable risk factor for resistant infection. We aimed to quantify the association between tobacco smoking history and antibiotic-resistant infection.

**Methods:**

We conducted a systematic review and meta-analysis of observational studies. Embase, PubMed, Scopus, and Web of Science were searched from database inception through 20 April 2026. Eligible studies reported a quantitative association between tobacco smoking history, defined as current or ever smoking compared with never smoking, and antibiotic-resistant bacterial infection. Adjusted odds ratios (ORs) were prioritized, with crude ORs used where adjusted estimates were unavailable or unusable. Risk of bias was assessed using ROBINS-E. Log ORs were pooled using a random-effects model with restricted maximum likelihood estimation. Prespecified subgroup analyses were conducted according to smoking definition, resistance group, and study design. Sensitivity analyses were restricted to adjusted estimates and excluded potentially influential estimates. Small-study effects were assessed using funnel plots and Egger’s test.

**Results:**

Forty-three studies, comprising more than 35,000 participants and contributing 43 estimates, were included. Most studies examined tuberculosis (TB)-related drug resistance (39/43, 91%); 23 studies used an ever-smoking definition, and 20 used a current-smoking definition. Tobacco smoking history was associated with higher odds of antibiotic-resistant infection (pooled OR = 1.66, 95% confidence interval [CI]: 1.33–2.07; I^2^ = 88.4%). Associations were observed for both current smoking (OR = 1.90, 95% CI: 1.26–2.87) and ever smoking (OR = 1.48, 95% CI: 1.15–1.90), as well as for both tuberculosis-related resistance (OR = 1.58, 95% CI: 1.26–1.98) and non-tuberculosis-related resistance (OR = 3.02, 95% CI: 1.23–7.41). The associations were directionally consistent across study designs; however, the cohort estimate was imprecise and did not reach statistical significance (case–control OR = 1.60, 95% CI: 1.01–2.55; cohort OR = 1.62, 95% CI: 0.64–4.09; cross-sectional OR = 1.64, 95% CI: 1.26–2.14). The overall association remained present in the analysis restricted to adjusted estimates (OR = 1.99, 95% CI: 1.56–2.56) and after excluding three influential studies (OR = 1.70, 95% CI: 1.37–2.12). Egger’s test did not indicate evidence of small-study effects (*p* = 0.55).

**Conclusion:**

Tobacco smoking history is associated with increased odds of antibiotic-resistant infection, with the evidence predominantly derived from studies of drug-resistant tuberculosis. Smoking cessation and tobacco-control interventions may be relevant to antimicrobial-resistance containment efforts, particularly in tuberculosis programs. Evidence concerning non-tuberculosis pathogens remains sparse, and substantial heterogeneity and residual confounding limit causal interpretation.

**Systematic review registration:**

PROSPERO Identifier CRD42024619290.

## Introduction

Antibiotic resistance is one of the defining public health challenges of the present era. In 2019, bacterial antimicrobial resistance was associated with an estimated 4.95 million deaths and was directly attributable to 1.27 million deaths globally (1). Although antimicrobial misuse and overuse are major drivers of resistance, the probability that an individual acquires, develops, or experiences an infection with a resistant organism is also shaped by host, clinical, environmental, and behavioral factors that affect exposure, colonization, immune competence, and treatment response (2).

Tobacco smoking remains a leading preventable cause of morbidity and mortality worldwide (3). Smoking damages the respiratory epithelium, impairs mucociliary clearance, alters alveolar macrophage and neutrophil function, dysregulates innate and adaptive immune responses, and modifies respiratory and oral microbial communities (4). These biological effects increase susceptibility to bacterial infection and may worsen clinical outcomes. Smoking is also an established risk factor for tuberculosis (TB) infection, active disease, and death (5, 6).

Several pathways could plausibly link smoking specifically to resistant infection. Impaired host clearance may prolong bacterial persistence during antibiotic exposure and increase opportunities for resistant subpopulations to emerge or be selected for (7, 8). Smoking-related comorbidity, delayed care seeking, treatment interruption, prior antimicrobial exposure, crowding, and socioeconomic disadvantage may also increase exposure to resistant pathogens (2, 4). In tuberculosis, smoking may interact with factors such as previous treatment, treatment adherence, and cavitary disease, all of which are relevant to drug resistance. For non-tuberculous bacterial infections, smoking has been associated with invasive bacterial disease and colonization patterns that could plausibly influence the acquisition of resistance (4).

Despite this biological and epidemiological plausibility, evidence linking smoking to antibiotic-resistant infection has accumulated piecemeal across pathogens, countries, and study designs. Existing systematic reviews have primarily focused on smoking and tuberculosis risk or on risk factors for drug-resistant tuberculosis, rather than quantifying the broader association between smoking history and antibiotic-resistant infection across bacterial pathogens (5, 6). Although our original review question used the broader term “antimicrobial resistance,” all eligible studies addressed antibacterial resistance. We therefore frame the present synthesis around antibiotic-resistant infection. This systematic review and meta-analysis aimed to estimate the association between tobacco smoking history and antibiotic-resistant infection and to examine whether this association varied according to smoking definition, resistance group, and study design.

## Methods

### Study design and registration

We conducted a systematic review and meta-analysis of observational studies and reported the study in accordance with the Preferred Reporting Items for Systematic Reviews and Meta-Analyses (PRISMA) 2020 statement (9) (see Additional file 1: PRISMA 2020 checklist). The protocol was registered with PROSPERO (CRD42024619290).

### Eligibility criteria

Eligible studies were observational studies, including case–control, cohort, and cross-sectional designs, that reported a quantitative association between tobacco smoking history and antibiotic-resistant bacterial infection. Using a population, exposure, comparator, outcome (PECO) framework, the population comprised individuals with bacterial infections or bacterial isolates for which antibiotic susceptibility or resistance status had been ascertained; the exposure was tobacco smoking history, defined as current or ever smoking; the comparator was never smoking; and the outcome was antibiotic-resistant infection or resistance among bacterial isolates. Studies were eligible if they reported an odds ratio (OR) with a 95% confidence interval (CI). We excluded studies that did not report a relevant resistance outcome or smoking exposure, studies without original human data, laboratory-based, animal, or purely mechanistic studies, conference abstracts with insufficient information, and reports from which an appropriate effect estimate could not be obtained.

### Information sources and search strategy

We searched Embase, PubMed, Scopus, and Web of Science from database inception through 20 April 2026. The search combined controlled vocabulary and free-text terms related to tobacco smoking, antimicrobial or antibiotic resistance, and infection, with the strategy adapted for each database. The complete search strategies are provided in Supplementary Text 1.

### Study selection

Search results were imported into Covidence for deduplication and screening. Duplicates were removed both automatically and manually where identified. Two reviewers independently screened titles and abstracts, followed by full-text assessment of potentially eligible reports. Disagreements were resolved through discussion.

### Data extraction

Two reviewers independently extracted data using a standardized data-extraction form. Disagreements were resolved through discussion. For each study, we extracted the first author, publication year, country, study design, population, sample size, the number and proportion of smokers, when reported, smoking comparison, resistance group, resistance outcome, causative organism, source of the effect estimate, OR and 95% CI, and information required for risk-of-bias assessment. One effect estimate was selected from each study. Where multiple eligible estimates were reported, we prioritized the most fully adjusted estimate corresponding to the prespecified smoking comparison and resistance outcome. Crude estimates were used when adjusted estimates were unavailable or could not be used.

### Effect measure and derivation

All effect estimates were analyzed as ORs on the natural logarithmic scale. The log OR was calculated as the natural logarithm of the reported point estimate. The standard error was derived from the reported 95% CI using the following formula: SE = [ln(upper limit) – ln(lower limit)] / (2 × 1.96). Reported point estimates were retained without modification. For studies with asymmetric CIs on the logarithmic scale, the CIs displayed in the forest plots may differ from the originally reported limits because they are reconstructed from the point estimate and derived standard error under the normal approximation. This reconstruction does not alter the reported point estimate used in the analysis.

### Risk-of-bias assessment

Two reviewers independently assessed risk of bias using the Risk Of Bias In Non-randomized Studies of Exposures (ROBINS-E) tool. Disagreements were resolved through discussion. Seven domains were considered: confounding, measurement of smoking exposure, selection of participants, post-exposure interventions, missing data, measurement of the resistance outcome, and selection of the reported result. The post-exposure intervention domain was generally considered not applicable to the included observational studies. Each study received an overall judgment of low risk of bias, some concerns, or high risk of bias.

### Statistical analysis

We pooled log ORs using random-effects meta-analysis estimated by restricted maximum likelihood (REML) because clinical and methodological heterogeneity was expected across populations, resistance outcomes, smoking definitions, study designs, and adjustment strategies. Results are presented as exponentiated ORs with 95% CIs. Heterogeneity was quantified with I^2^, tau^2^, H^2^, and Cochran’s Q test. Prespecified subgroup analyses were conducted according to smoking definition (current vs. never smoking and ever vs. never smoking), resistance group (tuberculosis-related vs. non-tuberculosis resistance), and study design (case–control, cohort, and cross-sectional). Between-subgroup differences were assessed using a Q-based test. Sensitivity analyses restricted the dataset to adjusted estimates and excluded three potentially influential studies identified after inspection of extreme or unusually precise estimates. Small-study effects were assessed through visual inspection of a funnel plot and Egger’s regression test. Analyses were conducted in StataNow version 19.5. A two-sided *p* value of less than 0.05 was considered statistically significant.

## Results

### Study selection

The search identified 3,775 records: 1,330 from Embase, 1,146 from Scopus, 878 from Web of Science, and 421 from PubMed. After the removal of 1,669 duplicates, including 12 identified manually and 1,657 identified in Covidence, 2,106 records were screened by title and abstract. Of these, 2,061 were excluded. Forty-five reports were assessed in full text, of which two were excluded because an appropriate effect estimate could not be obtained. In total, 43 studies were included in the systematic review and meta-analysis (10–52) (Figure 1).

**Figure 1:**
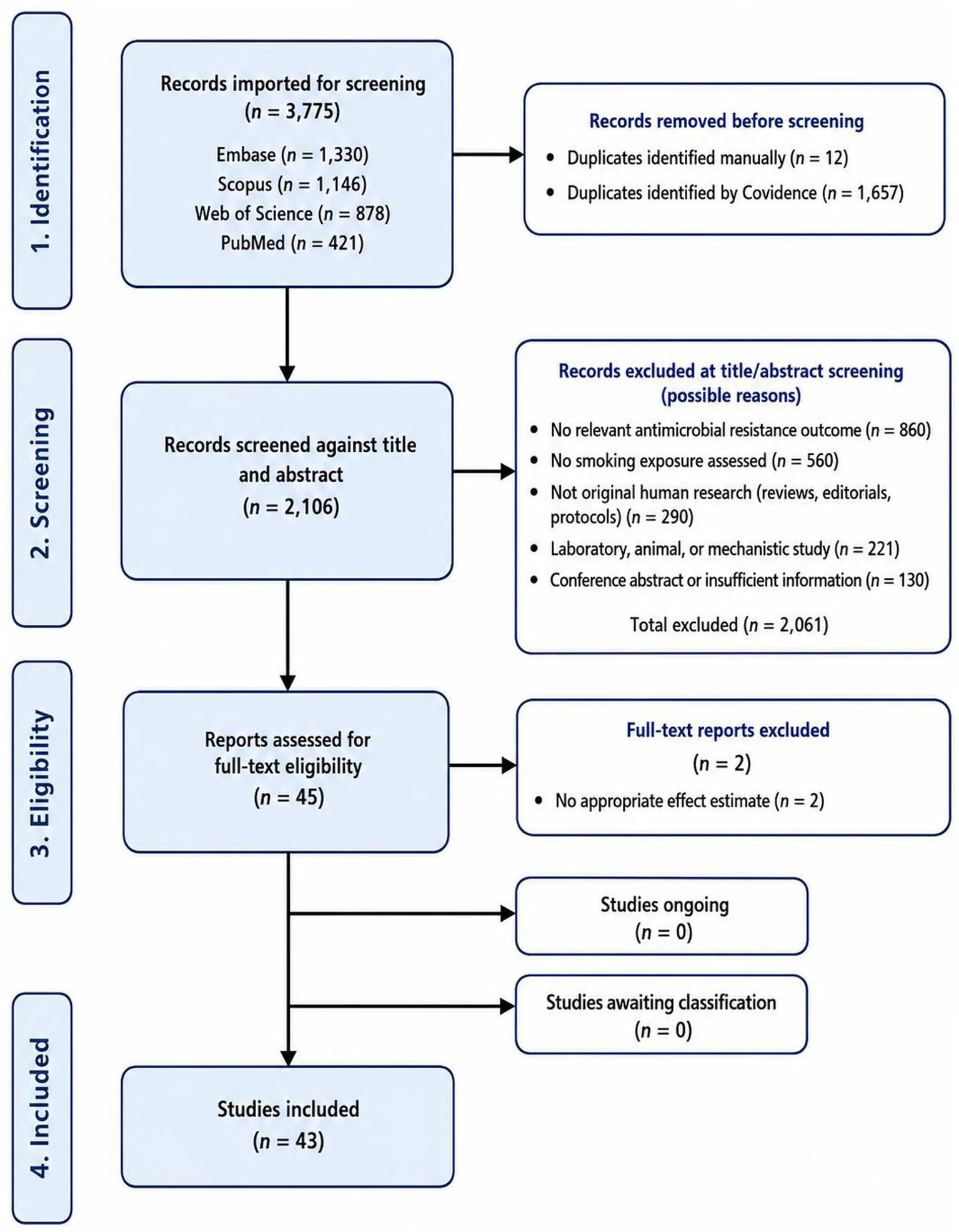
PRISMA 2020 flow diagram. Flow of records through identification, screening, eligibility, and inclusion. Of 3,775 records identified, 43 studies were included in the meta-analysis.

### Study characteristics

The 43 included studies were published between 2002 and 2023 and collectively enrolled more than 35,000 participants across Africa, Asia, Europe, South America, and multi-country settings (10–52). Twenty-three studies were cross-sectional (11, 12, 14, 19, 20, 24, 26, 28, 31, 32, 34–37, 40–42, 44, 46–49, 52), 17 were case–control studies (10, 13, 15, 17, 18, 21–23, 25, 27, 29, 30, 33, 39, 45, 50, 51), and three were cohort studies (16, 38, 43). The evidence base was dominated by tuberculosis: 39 studies examined tuberculosis-related resistance (10–21, 24–37, 39, 41–52), most commonly multidrug-resistant tuberculosis, whereas four studies examined non-tuberculosis resistance outcomes (22, 23, 38, 40). Twenty-three studies compared ever smokers with never smokers (10–14, 16, 20, 24–30, 32–35, 44, 46–49), and 20 compared current smokers with never smokers (15, 17–19, 21–23, 31, 36–43, 45, 50–52). Adjusted estimates were used for 28 studies (10, 11, 13, 14, 16–21, 26–28, 30, 32–34, 36, 39–45, 48, 51, 52), whereas crude estimates were used for 15 studies (12, 15, 22–25, 29, 31, 35, 37, 38, 46, 47, 49, 50). Summary characteristics are shown in Table 1; detailed study-level characteristics are provided in Supplementary Table S1.

**Table 1:** Essential characteristics of studies included in the systematic review and meta-analysis.

Study	Country	Design	Population, *N*	Smoking comparison	Resistance outcome and organism	Estimate source
Admassu et al. (10)	Ethiopia	Case–control	TB patients, 392	Ever vs. never smoking	MDR-TB; *Mycobacterium tuberculosis*	Adjusted
Ahmad et al. (11)	Pakistan	Cross-sectional	TB patients, 243	Ever vs. never smoking	Fluoroquinolone-resistant TB; *M. tuberculosis*	Adjusted
Al-Shahrani et al. (12)	Saudi Arabia	Cross-sectional	TB patients, 901	Ever vs. never smoking	MDR-TB and/or mono-resistance; *M. tuberculosis*	Crude
Ali et al. (13)	Sudan	Case–control	TB patients, 183	Ever vs. never smoking	MDR-TB; *M. tuberculosis*	Adjusted
An et al. (14)	China	Cross-sectional	TB patients, 12,661	Ever vs. never smoking	MDR-TB; *M. tuberculosis*	Adjusted
Balaji et al. (15)	India	Case–control	TB patients, 194	Current vs. never smoking	MDR-TB; *M. tuberculosis*	Crude
Dalton et al. (16)	Multi-country	Cohort	Pulmonary MDR-TB patients receiving second-line treatment, 214	Ever vs. never smoking	XDR-TB; *M. tuberculosis*	Adjusted
El Mahalli et al. (17)	Saudi Arabia	Case–control	Pulmonary TB patients, 181	Current vs. never smoking	Any drug-resistant TB; *M. tuberculosis*	Adjusted
Elduma et al. (18)	Sudan	Case–control	TB patients, 1,290	Current vs. never smoking	MDR-TB; *M. tuberculosis*	Adjusted
Welekidan et al. (19)	Ethiopia	Cross-sectional	TB patients, 227	Current vs. never smoking	MDR-TB; *M. tuberculosis*	Adjusted
Fregona et al. (20)	Brazil	Cross-sectional	TB patients, 1,578	Ever vs. never smoking	MDR-TB and/or mono-resistance; *M. tuberculosis*	Adjusted
Ignatyeva et al. (21)	Latvia, Lithuania and Estonia	Case–control	TB patients, 941	Current vs. never smoking	MDR-TB; *M. tuberculosis*	Adjusted
Isea-Peña et al. (22)	Spain	Case–control	Patients with invasive pneumococcal infection, 122	Current vs. never smoking	Levofloxacin-resistant *Streptococcus pneumoniae*	Crude
Jokinen et al. (23)	Finland	Case–control	Patients with *Staphylococcus aureus* bacteraemia, 504	Current vs. never smoking	MRSA bacteraemia; *S. aureus*	Crude
Junaid (24)	Pakistan	Cross-sectional	TB patients, 110	Ever vs. never smoking	MDR-TB; *M. tuberculosis*	Crude
Kadri et al. (25)	Indonesia	Case–control	TB patients, 292	Ever vs. never smoking	MDR-TB; *M. tuberculosis*	Crude
Lee et al. (26)	Korea	Cross-sectional	TB patients, 198	Ever vs. never smoking	MDR-TB; *M. tuberculosis*	Adjusted
Lema et al. (27)	Tanzania	Case–control	TB patients, 204	Ever vs. never smoking	MDR-TB; *M. tuberculosis*	Adjusted
Ma and Gao (28)	China	Cross-sectional	TB patients, 489	Ever vs. never smoking	Any drug-resistant TB; *M. tuberculosis*	Adjusted
Marahatta et al. (29)	Nepal	Case–control	TB patients, 110	Ever vs. never smoking	MDR-TB; *M. tuberculosis*	Crude
Marahatta et al. (30)	Nepal	Case–control	TB patients, 558	Ever vs. never smoking	MDR-TB; *M. tuberculosis*	Adjusted
Meriki et al. (31)	Cameroon	Cross-sectional	Adults attending TB and HIV care, 1,706	Current vs. never smoking	MDR-TB; *M. tuberculosis*	Crude
Mesfin et al. (32)	Ethiopia	Cross-sectional	TB patients, 226	Ever vs. never smoking	MDR-TB; *M. tuberculosis*	Adjusted
Min et al. (33)	Korea	Case–control	TB patients, 195	Ever vs. never smoking	MDR-TB; *M. tuberculosis*	Adjusted
Nguyen Thi Le Hang et al. (34)	Vietnam	Cross-sectional	TB patients, 546	Ever vs. never smoking	Any drug-resistant TB; *M. tuberculosis*	Adjusted
Olabiyi et al. (35)	Nigeria	Cross-sectional	TB patients, 1,203	Ever vs. never smoking	MDR-TB; *M. tuberculosis*	Crude
Otu et al. (36)	Nigeria	Cross-sectional	Pulmonary TB patients, 100	Current vs. never smoking	Any drug-resistant TB; *M. tuberculosis*	Adjusted
Prakash et al. (37)	India	Cross-sectional	TB patients, 274	Current vs. never smoking	MDR-TB; *M. tuberculosis*	Crude
Queiroz et al. (38)	Brazil	Cohort	Patients with duodenal ulcer or erosive gastritis, 97	Current vs. never smoking	*Helicobacter pylori* treatment failure	Crude
Rifat et al. (39)	Bangladesh	Case–control	TB patients, 1,000	Current vs. never smoking	MDR-TB; *M. tuberculosis*	Adjusted
Romero Nieto et al. (40)	Spain	Cross-sectional	Patients with community-acquired acute pyelonephritis, 367	Current vs. never smoking	ESBL-producing or resistant *Escherichia coli*/Enterobacteriaceae	Adjusted
Ruddy et al. (41)	Russia	Cross-sectional	TB patients, 600	Current vs. never smoking	Isoniazid-resistant TB; *M. tuberculosis*	Adjusted
Sahebi et al. (42)	Iran	Cross-sectional	HIV-negative, smear-positive pulmonary TB patients, 280	Current vs. never smoking	Any drug-resistant TB; *M. tuberculosis*	Adjusted
Salindri et al. (43)	Georgia	Cohort	MDR-TB patients, 267	Current vs. never smoking	Any drug-resistant TB; *M. tuberculosis*	Adjusted
Sambas et al. (44)	Saudi Arabia	Cross-sectional	TB patients, 158	Ever vs. never smoking	MDR-TB; *M. tuberculosis*	Adjusted
Sharma et al. (45)	India	Case–control	TB patients, 741	Current vs. never smoking	MDR-TB; *M. tuberculosis*	Adjusted
Sichen et al. (46)	China	Cross-sectional	TB patients, 395	Ever vs. never smoking	MDR-TB; *M. tuberculosis*	Crude
Sinha et al. (47)	India	Cross-sectional	Patients with clinically suspected TB, 721	Ever vs. never smoking	MDR-TB; *M. tuberculosis*	Crude
Skrahina et al. (48)	Belarus	Cross-sectional	TB patients, 1,420	Ever vs. never smoking	MDR-TB; *M. tuberculosis*	Adjusted
Souba Diandé et al. (49)	Burkina Faso	Cross-sectional	TB patients, 1,250	Ever vs. never smoking	Rifampicin-resistant TB; *M. tuberculosis*	Crude
Wijayanto et al. (50)	Indonesia	Case–control	TB patients, 90	Current vs. never smoking	MDR-TB; *M. tuberculosis*	Crude
Zhang et al. (51)	China	Case–control	TB patients, 578	Current vs. never smoking	MDR-TB; *M. tuberculosis*	Adjusted
Zhu et al. (52)	China	Cross-sectional	TB patients, 1,831	Current vs. never smoking	Any drug-resistant TB; *M. tuberculosis*	Adjusted

ESBL, extended-spectrum beta-lactamase; MDR-TB, multidrug-resistant tuberculosis; MRSA, methicillin-resistant *Staphylococcus aureus*; TB, tuberculosis; XDR-TB, extensively drug-resistant tuberculosis.

Resistance ascertainment was primarily based on microbiological or molecular drug-susceptibility testing, most often using sputum specimens or mycobacterial culture isolates in tuberculosis-related studies (10–21, 24–37, 39, 41–52). For TB-related outcomes, resistance markers most commonly involved rifampicin and isoniazid, with some studies also assessing other first-line drugs, fluoroquinolones, and second-line injectable agents (15, 16, 21, 33, 46, 48, 51, 52). The four non-tuberculosis studies used blood, cerebrospinal fluid, pericardial fluid, gastric biopsy, urine specimens, as well as blood culture isolates, to assess levofloxacin-resistant *Streptococcus pneumoniae* (22), methicillin-resistant *Staphylococcus aureus* bacteraemia (23), *Helicobacter pylori* treatment failure (38), and extended-spectrum beta-lactamase (ESBL)-producing or resistant *Escherichia coli* (40). Details of specimen sources, resistance ascertainment, organisms, and antimicrobial agents tested are provided in Supplementary Table S2.

### Risk of bias

Twenty studies were judged to have a high overall risk of bias (10, 12, 14, 15, 18, 22–27, 29, 31, 35, 37, 38, 46, 47, 49, 50), 20 had some concerns (11, 13, 17, 19–21, 28, 30, 32–34, 36, 39–45, 52), and three were judged to have a low risk of bias (16, 48, 51). High-risk judgments were commonly driven by uncontrolled confounding in studies contributing crude estimates. Additional concerns related to smoking-exposure measurement, participant selection, missing data, and outcome measurement. These concerns reflected the use of self-reported and often binary smoking classifications, limitations in study sampling, incomplete data, and variation in resistance ascertainment. Study-level domain and overall risk-of-bias judgments are provided in Supplementary Table S3.

### Primary meta-analysis

Tobacco smoking history was associated with higher odds of antibiotic-resistant infection (pooled OR = 1.66, 95% CI: 1.33–2.07; z = 4.46, *p* < 0.001; Figure 2). Between-study heterogeneity was substantial (I^2^ = 88.4%, tau^2^ = 0.36, H^2^ = 8.58; Q[42] = 165.21, *p* < 0.001), consistent with variation in pathogens, resistance outcomes, populations, study designs, and adjustment strategies. The funnel plot did not show strong evidence of asymmetry (Supplementary Figure S3), and Egger’s test did not indicate statistical evidence of small-study effects (*p* = 0.55).

**Figure 2:**
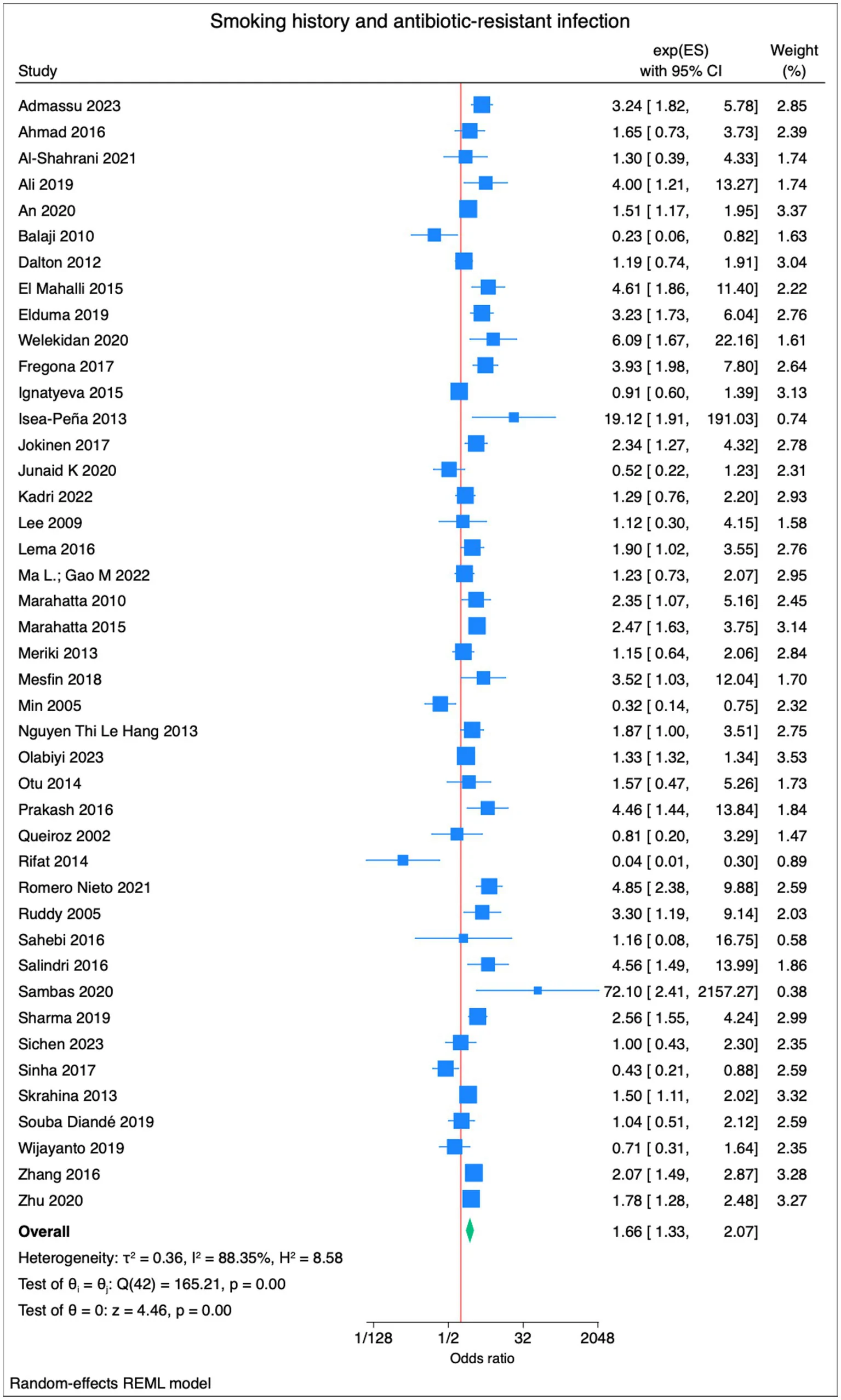
Forest plot of smoking history and antibiotic-resistant infection. Random-effects REML meta-analysis of 43 studies. Squares represent study ORs, with size proportional to weight; horizontal lines represent 95% CIs; and the diamond represents the pooled estimate. CIs in the forest plot are derived from each study’s point estimate and standard error under the normal approximation and may differ from originally reported limits for studies with asymmetric confidence intervals.

### Subgroup analyses

Associations were directionally consistent across prespecified subgroups. By smoking definition, the pooled OR was 1.90 (95% CI: 1.26–2.87) for current vs. never smoking and 1.48 (95% CI: 1.15–1.90) for ever vs. never smoking, with no evidence of a between-subgroup difference (Q_b_ *p* = 0.31; Figure 3). By resistance group, the pooled OR was 3.02 (95% CI: 1.23–7.41) for non-tuberculosis resistance and 1.58 (95% CI: 1.26–1.98) for tuberculosis-related resistance (Q_b_ *p* = 0.17; Figure 4). The non-tuberculosis estimate was based on only four studies and should be interpreted cautiously. By study design, estimates were closely similar for case–control studies (OR 1.60, 95% CI: 1.01–2.55), cohort studies (OR 1.62, 95% CI: 0.64–4.09), and cross-sectional studies (OR 1.64, 95% CI: 1.26–2.14), with no evidence of a between-subgroup difference (Q_b_ *p* = 0.996; Figure 5).

**Figure 3:**
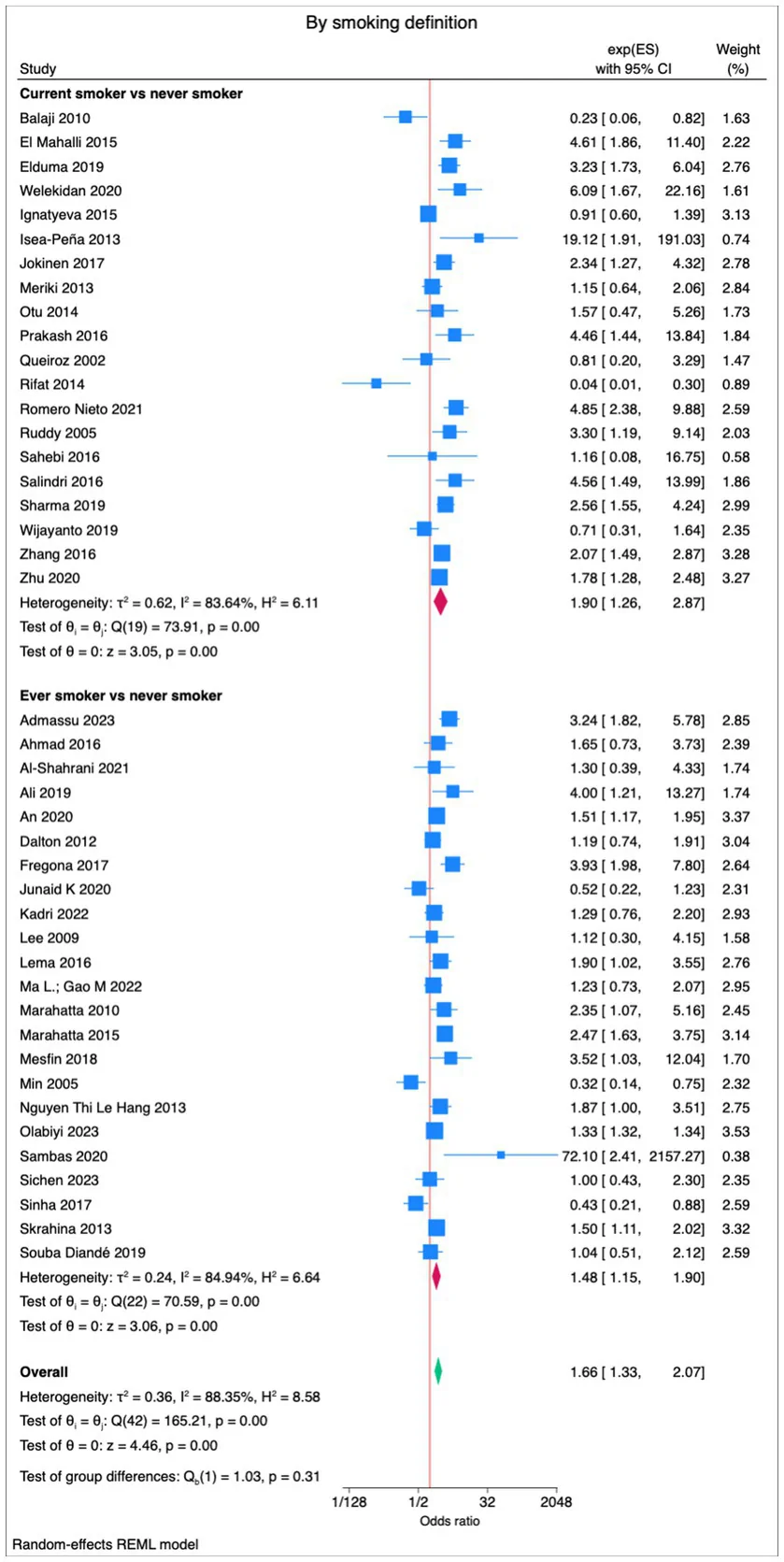
Subgroup meta-analysis by smoking definition. Random-effects REML subgroup analysis comparing current vs. never smoking and ever vs. never smoking.

**Figure 4:**
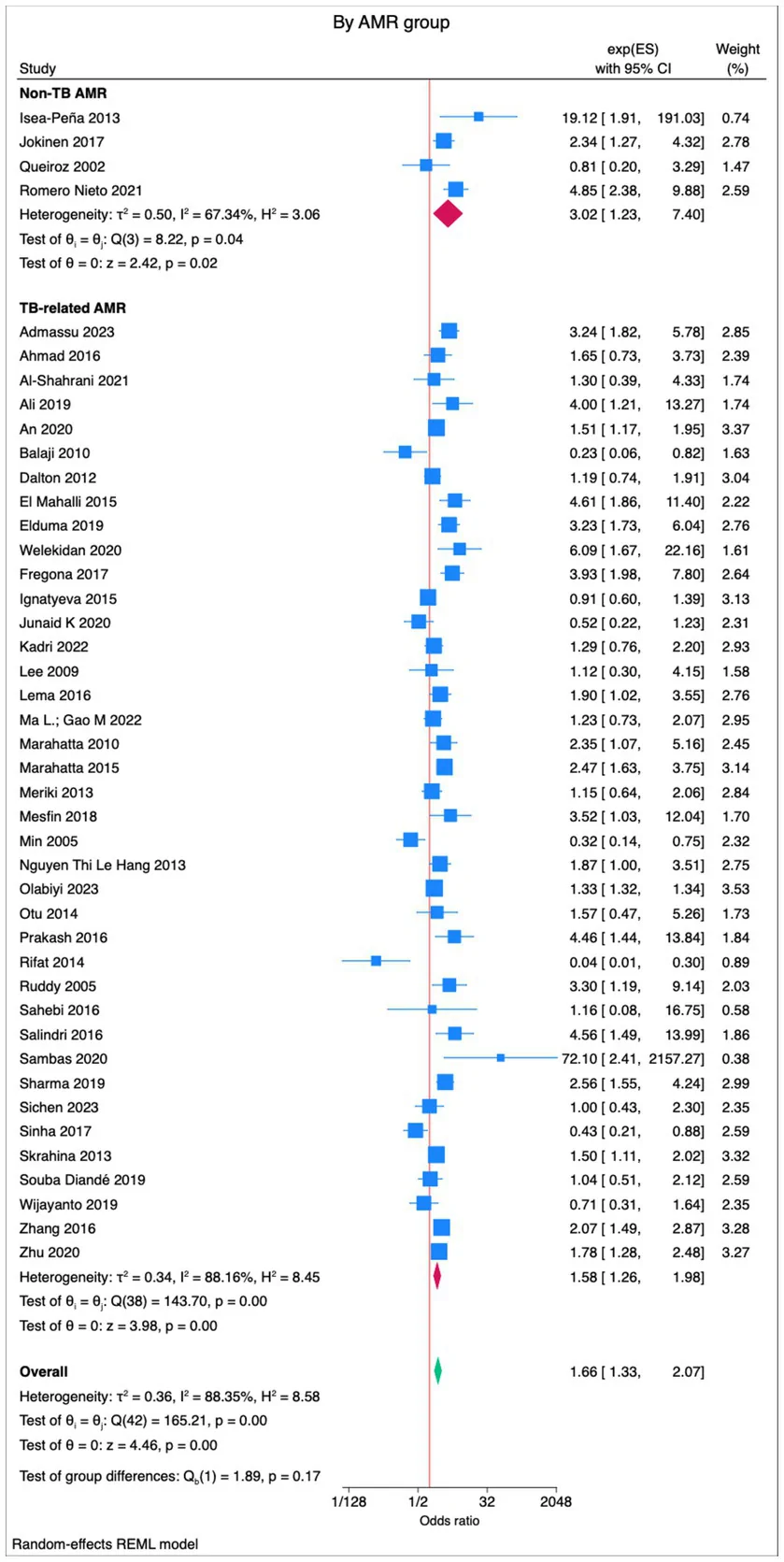
Subgroup meta-analysis by resistance group. Random-effects REML subgroup analysis comparing non-tuberculosis AMR and tuberculosis-related AMR. The non-tuberculosis subgroup included four studies and should be interpreted cautiously.

**Figure 5:**
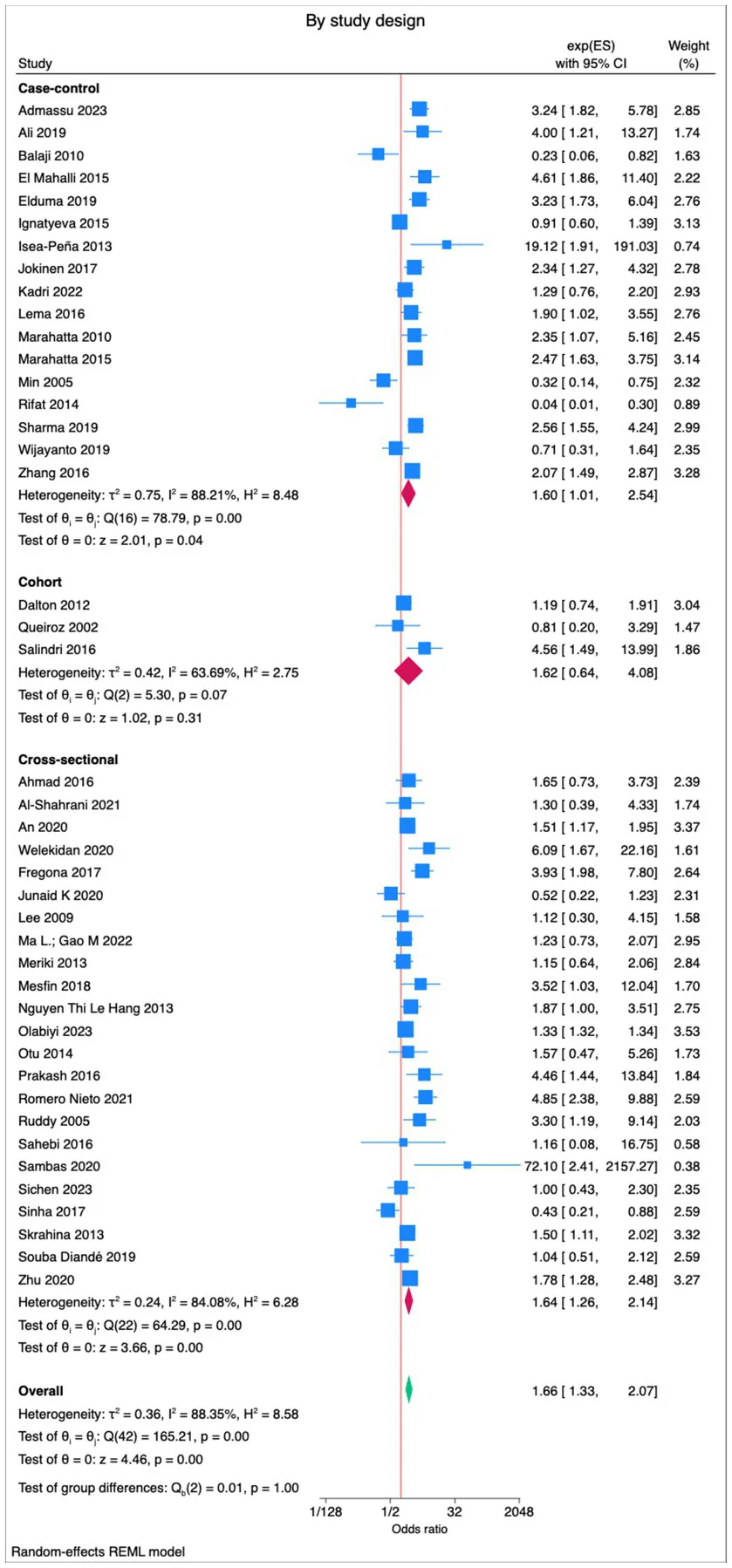
Subgroup meta-analysis by study design. Random-effects REML subgroup analysis comparing case–control, cohort, and cross-sectional studies.

### Sensitivity analyses

Restricting the analysis to the 28 adjusted estimates strengthened the association (OR = 1.99, 95% CI: 1.56–2.56; I^2^ = 77.1%; Supplementary Figure S1). Excluding three potentially influential estimates, Sambas 2020 (44), Olabiyi 2023 (35), and Rifat 2014 (39), produced a similar result (OR = 1.70, 95% CI: 1.37–2.12; I^2^ = 77.4%; Supplementary Figure S2). These findings indicate that the primary association was not driven by crude estimates or by the most extreme observations.

## Discussion

In this systematic review and meta-analysis of 43 observational studies, tobacco smoking history was associated with a 66% increase in the odds of antibiotic-resistant infection. The association was observed for both current and ever smoking definitions and was present in both tuberculosis-related and non-tuberculosis resistance groups, although the non-tuberculosis subgroup included only four studies and should be interpreted cautiously. By study design, the direction of association was broadly consistent across case–control, cohort, and cross-sectional studies, but the cohort estimate was imprecise and not statistically significant. The association strengthened when the analysis was restricted to adjusted estimates and remained similar after excluding three potentially influential studies, supporting the robustness of the overall finding. Only three cohort studies contributed to the cohort subgroup, compared with 17 case–control and 23 cross-sectional studies, providing substantially less information for the cohort synthesis. Importantly, the cohort point estimate (OR = 1.62) was nearly identical to the case–control (OR = 1.60) and cross-sectional (OR = 1.64) estimates; the non-significant result therefore reflects the wide confidence interval (95% CI: 0.64–4.09) rather than a materially different direction or magnitude of association.

This review extends previous evidence linking tobacco smoking to tuberculosis infection, disease, and poor outcomes (5, 6). The dominance of tuberculosis-related resistance among the included studies reflects both the global clinical importance of drug-resistant tuberculosis and the greater availability of epidemiological studies examining risk factors for multidrug-resistant tuberculosis (53). The finding should therefore be interpreted primarily as evidence for an association between smoking history and drug-resistant tuberculosis. However, the four non-tuberculosis studies, which addressed levofloxacin-resistant *Streptococcus pneumoniae* (22), methicillin-resistant *Staphylococcus aureus* bacteraemia (23), *Helicobacter pylori* treatment failure (38), and extended-spectrum beta-lactamase-producing or resistant *Escherichia coli* (40), suggest that the relationship may not be confined to tuberculosis, although this evidence remains too sparse to support firm conclusions. The non-tuberculosis estimate was imprecise and based on few studies; therefore, it should be seen as hypothesis-generating rather than definitive.

Related evidence on antibiotic exposure, although outside the outcome definition of this review, also provides useful context. Park et al. (54), using two nationally representative cohorts of adult men in South Korea, found no independent association between smoking history and subsequent broad-spectrum antibiotic use. By contrast, earlier studies reported higher subsequent antibacterial use or antibiotic prescribing among smokers (55, 56). These studies assessed antibiotic use or prescribing rather than microbiologically confirmed antibiotic resistance and therefore were not eligible for the present meta-analysis. Their mixed findings nevertheless reinforce the need for prospective studies that directly ascertain resistant infection while accounting for antibiotic exposure and other major confounders.

Several mechanisms could plausibly explain the observed association. Smoking damages epithelial barriers, disrupts mucociliary clearance, impairs macrophage and neutrophil function, and alters local microbial communities (4, 57–59). These effects can increase susceptibility to infection, prolong bacterial persistence, and potentially expose organisms to longer or repeated courses of antimicrobial therapy. In tuberculosis, smoking may contribute to delayed diagnosis, increased bacillary burden, cavitary disease, impaired treatment response, and treatment interruption (60), all of which are relevant to resistant disease. Behavioral and social pathways are also important: smoking is patterned by socioeconomic status, housing conditions, occupational exposure, alcohol use, comorbidity, and prior treatment history, and these factors may confound or mediate the association (61, 62).

Experimental evidence also raises the possibility of direct bacterial responses to tobacco-smoke constituents. *In vitro* exposure of *Pseudomonas aeruginosa* to cigarette smoke extract increased biofilm formation and levofloxacin resistance and upregulated multidrug-efflux-pump genes (59). Nicotine exposure has also been shown to enhance *Staphylococcus aureus* biofilm formation and alter bacterial regulatory and autolysis pathways, although that study did not demonstrate antibiotic resistance (63). Cigarette smoke contains multiple metals, including cadmium, lead, nickel, and zinc (64), and heavy-metal exposure can co-select for antibiotic resistance through co-resistance and cross-resistance, with additional evidence for stress-associated mutagenesis in experimental and environmental systems (65). These findings provide biological plausibility for direct bacterial adaptation under smoke-associated chemical stress, but they do not establish that this mechanism explains the epidemiological association observed here.

A host-mediated pathway is also plausible and is the more conservative interpretation of the present observational evidence. Smoking may create a favorable environment for infection, bacterial persistence, treatment failure, and repeated antimicrobial exposure, thereby increasing opportunities for resistant organisms to be acquired or selected without directly inducing resistance. The included studies assessed smoking history at the host level and did not measure bacterial exposure to nicotine, metals, or other smoke constituents, or changes in bacterial resistance mechanisms. We therefore cannot distinguish host-mediated susceptibility and selection from direct induction or co-selection of bacterial resistance, and the pooled association should not be interpreted as evidence that smoking itself causes bacteria to become antibiotic-resistant.

The adjusted-only analysis produced a larger pooled estimate than the primary analysis, indicating that inclusion of crude estimates did not account for the positive overall association. However, this difference should not be interpreted as evidence that adjustment strengthened a causal effect, because the adjusted and crude subsets comprised different studies and the covariates included in adjusted models varied considerably. Residual confounding therefore remains an important concern. Factors such as previous treatment, treatment adherence, HIV status, diabetes, alcohol use, socioeconomic position, and access to health care were inconsistently measured and controlled across studies. The pooled estimate should consequently be interpreted as an association rather than a causal effect.

This review has important public-health implications. If smoking contributes to the burden of antibiotic-resistant infection, especially drug-resistant tuberculosis, tobacco control and smoking-cessation support may have benefits beyond the prevention of cancer, cardiovascular disease, chronic respiratory disease, and drug-susceptible infection. Tuberculosis programs in high-burden settings could consider systematic identification of smoking status and integration of cessation support into treatment pathways. More broadly, antimicrobial-resistance strategies often focus on antimicrobial prescribing, infection prevention, diagnostics, and surveillance; our findings suggest that behavioral risk reduction may also deserve attention within a comprehensive resistance-containment agenda.

The review also highlights important gaps. Evidence for non-tuberculous antibiotic-resistant infections is sparse. Future studies should examine smoking and resistance in urinary tract infection, bloodstream infection, pneumonia, wound infection, and colonization with resistant organisms. Prospective designs with clear temporality, harmonized smoking definitions, dose–response assessment, former-smoker categories, biochemical validation where feasible, and robust confounder adjustment are needed. Mechanistic studies should explore whether smoking-related immune impairment, microbiome changes, pharmacokinetic effects, or treatment adherence pathways explain the observed association.

The strengths of this review include a broad multi-database search, duplicate screening, extraction of a single effect estimate per study, prioritization of adjusted estimates, prespecified subgroup and sensitivity analyses, and formal risk-of-bias assessment. Important limitations should also be acknowledged. Heterogeneity was high and was not fully explained by smoking definition, resistance group, or study design. Most included studies were cross-sectional or case–control, limiting causal inference and, for some studies, temporality. Smoking exposure was usually self-reported and often reduced to a binary exposure, without information on intensity, duration, pack-years, recency, or cessation. Fifteen studies contributed crude estimates. Some studies reported asymmetric confidence intervals on the log scale; we retained reported point estimates and derived standard errors from the reported intervals, so displayed forest-plot CIs may differ from original published limits for those studies. Finally, the evidence base was heavily weighted toward tuberculosis, limiting generalizability to other pathogens.

## Conclusion

Tobacco smoking history is associated with higher odds of antibiotic-resistant infection, with evidence driven predominantly by drug-resistant tuberculosis and robust to key sensitivity analyses. Smoking is a modifiable exposure and may represent an under-recognized behavioral contributor to the burden of resistant infection. Integration of smoking-cessation interventions into tuberculosis and antimicrobial-resistance control programs merits consideration, while better prospective and pathogen-diverse evidence is needed to clarify causality and generalizability beyond tuberculosis.

## Data Availability

The original contributions presented in the study are included in the article/Supplementary material, further inquiries can be directed to the corresponding author.
