# Supplementary material for: Tobacco smoking history and antibiotic-resistant infection: a systematic review and meta-analysis
Source: Front Public Health. 2026 Oct 7;14:1935025. doi: 10.3389/fpubh.2026.1935025 (PMC13688436; doi:10.3389/fpubh.2026.1935025)
Supplement: Supplementary file 1 [file Supplementary_file_1.docx]

Additional file 1: Supplementary appendix

Supplementary Text 1. Search strategy

Search conducted on 20 April 2026. The search strategies below were extracted from the submitted search-strategy file.

Ovid Embase

Cigarette smoking.mp. or 'Cigarette Smoking'/
Smoking.mp. or 'Smoking'/
'Tobacco Use'/
Cigarettes.mp. or 'Cigarettes'/
Tobacco products.mp. or 'Tobacco Products'/
Nicotine dependence.mp. or 'Nicotine Dependence'/
Tobacco smoking.mp. or 'Tobacco Smoking'/
Passive Smoking/ or 'Secondhand Smoke'/
Smoker/ or 'Former Smoker'/ or 'ex-smoker'/
1 or 2 or 3 or 4 or 5 or 6 or 7 or 8 or 9
Antimicrobial resistance.mp. or Drug Resistance, Microbial/
Antibiotic resistance.mp. or Drug Resistance, Bacterial/
Multidrug resistance.mp. or Drug Resistance, Multiple/
MDR.mp. or Multiple Drug Resistance/
XDR.mp. or Extensive Drug Resistance/
PDR.mp. or Pan-Drug Resistance/
Beta-Lactam Resistance/ or Beta-Lactamase/
Vancomycin Resistance/
Methicillin Resistance/
Carbapenem Resistance/
ESBL.mp. or 'Extended-Spectrum Beta-Lactamase'/
11 or 12 or 13 or 14 or 15 or 16 or 17 or 18 or 19 or 20 or 21
Infections.mp. or Infection/
Bacterial infections.mp. or Bacterial Infections/
Viral infections.mp. or Virus Diseases/
Fungal infections.mp. or Mycoses/
Parasitic infections.mp. or Parasitic Diseases/
Pneumonia.mp. or Pneumonia/
Tuberculosis.mp. or Tuberculosis/
HIV.mp. or HIV Infections/
AIDS.mp. or Acquired Immunodeficiency Syndrome/
Influenza.mp. or Influenza, Human/
Respiratory tract infections.mp. or Respiratory Tract Infections/
Urinary tract infections.mp. or Urinary Tract Infections/
Sepsis.mp. or Sepsis/
Malaria.mp. or Malaria/
Gastroenteritis.mp. or Gastroenteritis/
Hepatitis.mp. or Hepatitis, Viral, Human/
Skin infections.mp. or Skin Diseases, Infectious/
Meningitis.mp. or Meningitis/
Otitis media.mp. or Otitis Media/
Sinusitis.mp. or Sinusitis/
Bronchitis.mp. or Bronchitis/
Pharyngitis.mp. or Pharyngitis/
Gastrointestinal infections.mp. or Gastrointestinal Infections/
23 or 24 or 25 or 26 or 27 or 28 or 29 or 30 or 31 or 32 or 33 or 34
or 35 or 36 or 37 or 38 or 39 or 40 or 41 or 42 or 43 or 44 or 45
10 and 22 and 46

Web of Science

TS=("cigarette smoking" OR "smoking" OR "tobacco use" OR
"cigarettes" OR "tobacco products" OR "nicotine dependence" OR
"tobacco smoking" OR "passive smoking" OR "secondhand smoke"
OR "former smoker" OR "ex-smoker") AND TS=("antimicrobial
resistance" OR "drug resistance, microbial" OR "antibiotic
resistance" OR "multidrug resistance" OR "MDR" OR "XDR" OR
"PDR" OR "beta-lactam resistance" OR "beta-lactamase" OR
"vancomycin resistance" OR "methicillin resistance" OR
"carbapenem resistance" OR "ESBL" OR "extended-spectrum beta-
lactamase") AND TS=("infections" OR "infection" OR "bacterial
infections" OR "viral infections" OR "fungal infections" OR
"parasitic infections" OR "pneumonia" OR "tuberculosis" OR
"HIV" OR "AIDS" OR "influenza" OR "respiratory tract infections"
OR "urinary tract infections" OR "sepsis" OR "malaria" OR
"gastroenteritis" OR "hepatitis" OR "skin infections" OR
"meningitis" OR "otitis media" OR "sinusitis" OR "bronchitis" OR
"pharyngitis" OR "gastrointestinal infections")

Scopus

( TITLE-ABS-KEY ( "cigarette smoking" OR "smoking" OR
"tobacco use" OR "cigarettes" OR "tobacco products" OR "nicotine
dependence" OR "tobacco smoking" OR "passive smoking" OR
"secondhand smoke" OR "former smoker" OR "ex-smoker" ) )
AND ( TITLE-ABS-KEY ( "antimicrobial resistance" OR "drug
resistance, microbial" OR "antibiotic resistance" OR "multidrug
resistance" OR "MDR" OR "XDR" OR "PDR" OR "beta-lactam
resistance" OR "beta-lactamase" OR "vancomycin resistance" OR
"methicillin resistance" OR "carbapenem resistance" OR "ESBL"
OR "extended-spectrum beta-lactamase" ) ) AND ( TITLE-ABS-
KEY ( "infections" OR "infection" OR "bacterial infections" OR
"viral infections" OR "fungal infections" OR "parasitic infections"
OR "pneumonia" OR "tuberculosis" OR "HIV" OR "AIDS" OR
"influenza" OR "respiratory tract infections" OR "urinary tract
infections" OR "sepsis" OR "malaria" OR "gastroenteritis" OR
"hepatitis" OR "skin infections" OR "meningitis" OR "otitis media"
OR "sinusitis" OR "bronchitis" OR "pharyngitis" OR
"gastrointestinal infections" ) )

PubMed

(("Cigarette Smoking"[MeSH Terms] OR "Smoking"[MeSH
Terms] OR "Tobacco Use"[MeSH Terms] OR "Tobacco
Products"[MeSH Terms] OR "Tobacco Use Disorder"[MeSH
Terms] OR "Smoking Cessation"[MeSH Terms] OR
"Tobacco"[MeSH Terms] OR "Nicotine"[MeSH Terms] OR
"cigarette smoking"[All Fields] OR "smoking"[All Fields] OR
"tobacco use"[All Fields] OR "cigarettes"[All Fields] OR "tobacco
products"[All Fields] OR "nicotine dependence"[All Fields] OR
"tobacco smoking"[All Fields] OR "passive smoking"[All Fields]
OR "secondhand smoke"[All Fields] OR "former smoker"[All
Fields] OR "ex-smoker"[All Fields])) AND ("Drug Resistance,
Microbial"[MeSH Terms] OR "Drug Resistance, Bacterial"[MeSH
Terms] OR "Multiple Drug Resistance"[MeSH Terms] OR "Beta-
Lactam Resistance"[All Fields] OR "Vancomycin Resistance"[All
Fields] OR "Methicillin Resistance"[All Fields] OR
"Carbapenems"[MeSH Terms] OR "Extended-Spectrum Beta-
Lactamases"[All Fields] OR "antimicrobial resistance"[All Fields]
OR "antibiotic resistance"[All Fields] OR "multidrug
resistance"[All Fields] OR "MDR"[All Fields] OR "XDR"[All
Fields] OR "PDR"[All Fields] OR "beta-lactam resistance"[All
Fields] OR "vancomycin resistance"[All Fields] OR "methicillin
resistance"[All Fields] OR "carbapenem resistance"[All Fields] OR
"ESBL"[All Fields] OR "extended-spectrum beta-lactamase"[All
Fields])) AND ("Infections"[MeSH Terms] OR "Bacterial
Infections"[MeSH Terms] OR "Mycoses"[MeSH Terms] OR
"Parasitic Diseases"[MeSH Terms] OR "Pneumonia"[MeSH Terms]
OR "Tuberculosis"[MeSH Terms] OR "HIV Infections"[MeSH
Terms] OR "Acquired Immunodeficiency Syndrome"[MeSH
Terms] OR "Influenza, Human"[MeSH Terms] OR "Respiratory
Tract Infections"[MeSH Terms] OR "Urinary Tract
Infections"[MeSH Terms] OR "Sepsis"[MeSH Terms] OR
"Malaria"[MeSH Terms] OR "Gastroenteritis"[MeSH Terms] OR
"Hepatitis, Viral, Human"[MeSH Terms] OR "Skin Diseases,
Infectious"[MeSH Terms] OR "Meningitis"[MeSH Terms] OR
"Otitis Media"[MeSH Terms] OR "Sinusitis"[MeSH Terms] OR
"Bronchitis"[MeSH Terms] OR "Pharyngitis"[MeSH Terms] OR
"Gastrointestinal Diseases"[MeSH Terms] OR "infections"[All
Fields] OR "infection"[All Fields] OR "bacterial infections"[All
Fields] OR "viral infections"[All Fields] OR "fungal infections"[All
Fields] OR "parasitic infections"[All Fields] OR "pneumonia"[All
Fields] OR "tuberculosis"[All Fields] OR "HIV"[All Fields] OR
"AIDS"[All Fields] OR "influenza"[All Fields] OR "respiratory
tract infections"[All Fields] OR "urinary tract infections"[All
Fields] OR "sepsis"[All Fields] OR "malaria"[All Fields] OR
"gastroenteritis"[All Fields] OR "hepatitis"[All Fields] OR "skin
infections"[All Fields] OR "meningitis"[All Fields] OR "otitis
media"[All Fields] OR "sinusitis"[All Fields] OR "bronchitis"[All
Fields] OR "pharyngitis"[All Fields] OR "gastrointestinal
infections"[All Fields]))

Additional file 1: PRISMA 2020 Checklist

Manuscript: Tobacco smoking history and antibiotic-resistant infection: a systematic review and meta-analysis

Completed using the submitted manuscript and PRISMA checklist template.

| **Section and Topic** | **Item #** | **Checklist item** | **Location where item is reported** |
| --- | --- | --- | --- |
| **TITLE** |  |  |  |
| **Title** | **1** | **Identify the report as a systematic review.** | **Main text, Title** |
| **ABSTRACT** |  |  |  |
| **Abstract** | **2** | **See the PRISMA 2020 for Abstracts checklist.** | **Main text, Abstract** |
| **INTRODUCTION** |  |  |  |
| Rationale | 3 | Describe the rationale for the review in the context of existing knowledge. | Main text, Introduction, paragraphs 1–4 |
| Objectives | 4 | Provide an explicit statement of the objective(s) or question(s) the review addresses. | Main text, Introduction, final paragraph |
| **METHODS** |  |  |  |
| Eligibility criteria | 5 | Specify the inclusion and exclusion criteria for the review and how studies were grouped for the syntheses. | Main text, Methods—Eligibility criteria |
| Information sources | 6 | Specify all databases, registers, websites, organisations, reference lists and other sources searched or consulted to identify studies. Specify the date when each source was last searched or consulted. | Main text, Methods—Information sources and search strategy |
| Search strategy | 7 | Present the full search strategies for all databases, registers and websites, including any filters and limits used. | Main text, Methods—Information sources and search strategy; Additional file 1, Supplementary Text 1 |
| Selection process | 8 | Specify the methods used to decide whether a study met the inclusion criteria of the review, including how many reviewers screened each record and each report retrieved, whether they worked independently, and if applicable, details of automation tools used in the process. | Main text, Methods—Study selection |
| Data collection process | 9 | Specify the methods used to collect data from reports, including how many reviewers collected data from each report, whether they worked independently, any processes for obtaining or confirming data from study investigators, and if applicable, details of automation tools used in the process. | Main text, Methods—Data extraction |
| Data items | 10a | List and define all outcomes for which data were sought. Specify whether all results that were compatible with each outcome domain in each study were sought, and if not, the methods used to decide which results to collect. | Main text, Methods—Eligibility criteria and Data extraction |
| Data items | 10b | List and define all other variables for which data were sought. Describe any assumptions made about any missing or unclear information. | Main text, Methods—Data extraction |
| Study risk of bias assessment | 11 | Specify the methods used to assess risk of bias in the included studies, including details of the tool(s) used, how many reviewers assessed each study and whether they worked independently, and if applicable, details of automation tools used in the process. | Main text, Methods—Risk-of-bias assessment |
| Effect measures | 12 | Specify for each outcome the effect measure(s) used in the synthesis or presentation of results. | Main text, Methods—Effect measure and derivation |
| Synthesis methods | 13a | Describe the processes used to decide which studies were eligible for each synthesis. | Main text, Methods—Eligibility criteria and Statistical analysis |
| Synthesis methods | 13b | Describe any methods required to prepare the data for presentation or synthesis, such as handling of missing summary statistics, or data conversions. | Main text, Methods—Effect measure and derivation |
| Synthesis methods | 13c | Describe any methods used to tabulate or visually display results of individual studies and syntheses. | Main text, Methods—Statistical analysis; Figures 2–5; Supplementary Figures S1–S3 |
| Synthesis methods | 13d | Describe any methods used to synthesize results and provide a rationale for the choice(s). If meta-analysis was performed, describe the model(s), method(s) used to identify the presence and extent of statistical heterogeneity, and software package(s) used. | Main text, Methods—Statistical analysis |
| Synthesis methods | 13e | Describe any methods used to explore possible causes of heterogeneity among study results. | Main text, Methods—Statistical analysis |
| Synthesis methods | 13f | Describe any sensitivity analyses conducted to assess robustness of the synthesized results. | Main text, Methods—Statistical analysis |
| Reporting bias assessment | 14 | Describe any methods used to assess risk of bias due to missing results in a synthesis, arising from reporting biases. | Main text, Methods—Statistical analysis |
| Certainty assessment | 15 | Describe any methods used to assess certainty or confidence in the body of evidence for an outcome. | **Not assessed** |
| **RESULTS** |  |  |  |
| Study selection | 16a | Describe the results of the search and selection process, from the number of records identified in the search to the number of studies included in the review, ideally using a flow diagram. | Main text, Results—Study selection; Figure 1 |
| Study selection | 16b | Cite studies that might appear to meet the inclusion criteria, but which were excluded, and explain why they were excluded. | Main text, Results—Study selection; Figure 1 |
| Study characteristics | 17 | Cite each included study and present its characteristics. | Main text, Results—Study characteristics; Table 1; Supplementary Tables S1–S2 |
| Risk of bias in studies | 18 | Present assessments of risk of bias for each included study. | Main text, Results—Risk of bias; Supplementary Table S3 |
| Results of individual studies | 19 | For all outcomes, present, for each study: summary statistics for each group where appropriate and an effect estimate and its precision, ideally using structured tables or plots. | Main text, Results—Primary meta-analysis, Subgroup analyses and Sensitivity analyses; Figures 2–5; Supplementary Figures S1–S2 |
| Results of syntheses | 20a | For each synthesis, briefly summarise the characteristics and risk of bias among contributing studies. | Main text, Results—Study characteristics, Risk of bias, Primary meta-analysis and Subgroup analyses |
| Results of syntheses | 20b | Present results of all statistical syntheses conducted. If meta-analysis was done, present for each the summary estimate and its precision and measures of statistical heterogeneity. If comparing groups, describe the direction of the effect. | Main text, Results—Primary meta-analysis, Subgroup analyses and Sensitivity analyses; Figures 2–5; Supplementary Figures S1–S2 |
| Results of syntheses | 20c | Present results of all investigations of possible causes of heterogeneity among study results. | Main text, Results—Subgroup analyses; Figures 3–5 |
| Results of syntheses | 20d | Present results of all sensitivity analyses conducted to assess the robustness of the synthesized results. | Main text, Results—Sensitivity analyses; Supplementary Figures S1–S2 |
| Reporting biases | 21 | Present assessments of risk of bias due to missing results, arising from reporting biases, for each synthesis assessed. | Main text, Results—Primary meta-analysis; Supplementary Figure S3 |
| Certainty of evidence | 22 | Present assessments of certainty or confidence in the body of evidence for each outcome assessed. | **Not assessed** |
| **DISCUSSION** |  |  |  |
| **Discussion** | **23a** | **Provide a general interpretation of the results in the context of other evidence.** | **Main text, Discussion, paragraphs 1–4** |
| **Discussion** | **23b** | **Discuss any limitations of the evidence included in the review.** | **Main text, Discussion—limitations paragraph** |
| **Discussion** | **23c** | **Discuss any limitations of the review processes used.** | **Main text, Discussion—limitations paragraph** |
| **Discussion** | **23d** | **Discuss implications of the results for practice, policy, and future research.** | **Main text, Discussion—public-health implications and research-gaps paragraphs** |
| **OTHER INFORMATION** |  |  |  |
| Registration and protocol | 24a | Provide registration information for the review, including register name and registration number, or state that the review was not registered. | Main text, Abstract—Systematic review registration; Methods—Study design and registration |
| Registration and protocol | 24b | Indicate where the review protocol can be accessed, or state that a protocol was not prepared. | Main text, Methods—Study design and registration |
| Registration and protocol | 24c | Describe and explain any amendments to information provided at registration or in the protocol. | Not applicable / no amendments reported |
| Support | 25 | Describe sources of financial or non-financial support for the review, and the role of the funders or sponsors in the review. | Main text, Declarations—Funding |
| Competing interests | 26 | Declare any competing interests of review authors. | Main text, Declarations—Competing interests |
| Availability of data, code and other materials | 27 | Report which of the following are publicly available and where they can be found: template data collection forms; data extracted from included studies; data used for all analyses; analytic code; any other materials used in the review. | Data/materials included in article and supplementary files; no separate code availability statement |

Supplementary Table S1. Detailed characteristics of included studies

AMR, antimicrobial resistance; CI, confidence interval; ESBL, extended-spectrum beta-lactamase; MDR-TB, multidrug-resistant tuberculosis; MRSA, methicillin-resistant Staphylococcus aureus; OR, odds ratio; XDR-TB, extensively drug-resistant tuberculosis.

| **Study** | **Country** | **Design** | **Population** | **N** | **Smokers, n (%)** | **Smoking comparison** | **AMR group** | **AMR outcome** | **Organism** | **Estimate source** | **OR (95% CI)** |
| --- | --- | --- | --- | --- | --- | --- | --- | --- | --- | --- | --- |
| **Admassu 2023** | Ethiopia | Case-control | TB patients | 392 | 48 (12.2%) | Ever smoker vs never smoker | TB-related AMR | MDR-TB | *Mycobacterium tuberculosis* | Adjusted | 3.24 (2.17–6.91) |
| **Ahmad 2016** | Pakistan | Cross-sectional | TB patients | 243 | 31 (12.8%) | Ever smoker vs never smoker | TB-related AMR | Fluoroquinolone-resistant TB | *Mycobacterium tuberculosis* | Adjusted | 1.65 (0.73–3.74) |
| **Al-Shahrani 2021** | Saudi Arabia | Cross-sectional | TB patients | 901 | 29 (3.2%) | Ever smoker vs never smoker | TB-related AMR | MDR-TB and/or mono-resistance | *Mycobacterium tuberculosis* | Crude | 1.30 (0.39–4.30) |
| **Ali 2019** | Sudan | Case-control | TB patients | 183 | 91 (49.7%) | Ever smoker vs never smoker | TB-related AMR | MDR-TB | *Mycobacterium tuberculosis* | Adjusted | 4.00 (1.20–13.20) |
| **An 2020** | China | Cross-sectional | TB patients | 12,661 | 792 (6.3%) | Ever smoker vs never smoker | TB-related AMR | MDR-TB | *Mycobacterium tuberculosis* | Adjusted | 1.51 (1.17–1.95) |
| **Balaji 2010** | India | Case-control | TB patients | 194 | Not reported | Current smoker vs never smoker | TB-related AMR | MDR-TB | *Mycobacterium tuberculosis* | Crude | 0.23 (0.06–0.81) |
| **Dalton 2012** | Multi-country (Estonia, Latvia, Peru, Philippines, Russia, South Africa, South Korea, Thailand) | Cohort | Pulmonary MDR-TB patients receiving second-line treatment | 214 | Not reported | Ever smoker vs never smoker | TB-related AMR | XDR-TB | *Mycobacterium tuberculosis* | Adjusted | 1.19 (0.74–1.91) |
| **El Mahalli 2015** | Saudi Arabia | Case-control | Pulmonary TB patients | 181 | Not reported | Current smoker vs never smoker | TB-related AMR | Any drug-resistant TB | *Mycobacterium tuberculosis* | Adjusted | 4.61 (1.86–11.38) |
| **Elduma 2019** | Sudan | Case-control | TB patients | 1,290 | Not reported | Current smoker vs never smoker | TB-related AMR | MDR-TB | *Mycobacterium tuberculosis* | Adjusted | 3.23 (1.73–6.04) |
| **Welekidan 2020** | Ethiopia | Cross-sectional | TB patients | 227 | 19 (8.4%) | Current smoker vs never smoker | TB-related AMR | MDR-TB | *Mycobacterium tuberculosis* | Adjusted | 6.09 (1.70–22.50) |
| **Fregona 2017** | Brazil | Cross-sectional | TB patients | 1,578 | Not reported | Ever smoker vs never smoker | TB-related AMR | MDR-TB and/or mono-resistance | *Mycobacterium tuberculosis* | Adjusted | 3.93 (1.98–7.79) |
| **Ignatyeva 2015** | Latvia, Lithuania, and Estonia | Case-control | TB patients | 941 | 681 (72.4%) | Current smoker vs never smoker | TB-related AMR | MDR-TB | *Mycobacterium tuberculosis* | Adjusted | 0.91 (0.60–1.39) |
| **Isea-Peña 2013** | Spain | Case-control | Invasive pneumococcal infections | 122 | 58 (47.5%) | Current smoker vs never smoker | Non-TB AMR | Levofloxacin-resistant Streptococcus pneumoniae | *Streptococcus pneumoniae* | Crude | 19.12 (3.56–355.40) |
| **Jokinen 2017** | Finland | Case-control | Staphylococcus aureus bacteremia | 504 | 94 (18.7%) | Current smoker vs never smoker | Non-TB AMR | MRSA bacteraemia | *Staphylococcus aureus* | Crude | 2.34 (1.27–4.32) |
| **Junaid K 2020** | Pakistan | Cross-sectional | TB patients | 110 | 61 (55.5%) | Ever smoker vs never smoker | TB-related AMR | MDR-TB | *Mycobacterium tuberculosis* | Crude | 0.52 (0.22–1.23) |
| **Kadri 2022** | Indonesia | Case-control | TB patients | 292 | 126 (43.2%) | Ever smoker vs never smoker | TB-related AMR | MDR-TB | *Mycobacterium tuberculosis* | Crude | 1.29 (0.76–2.21) |
| **Lee 2009** | Korea | Cross-sectional | TB patients | 198 | 65 (32.8%) | Ever smoker vs never smoker | TB-related AMR | MDR-TB | *Mycobacterium tuberculosis* | Adjusted | 1.12 (0.30–4.12) |
| **Lema 2016** | Tanzania | Case-control | TB patients | 204 | 107 (52.5%) | Ever smoker vs never smoker | TB-related AMR | MDR-TB | *Mycobacterium tuberculosis* | Adjusted | 1.90 (1.00–3.50) |
| **Ma L.; Gao M 2022** | China | Cross-sectional | TB patients | 489 | 52 (10.6%) | Ever smoker vs never smoker | TB-related AMR | Any drug-resistant TB | *Mycobacterium tuberculosis* | Adjusted | 1.23 (1.02–2.89) |
| **Marahatta 2010** | Nepal | Case-control | TB patients | 110 | 43 (39.1%) | Ever smoker vs never smoker | TB-related AMR | MDR-TB | *Mycobacterium tuberculosis* | Crude | 2.35 (1.07–5.16) |
| **Marahatta 2015** | Nepal | Case-control | TB patients | 558 | 144 (25.8%) | Ever smoker vs never smoker | TB-related AMR | MDR-TB | *Mycobacterium tuberculosis* | Adjusted | 2.47 (1.21–2.79) |
| **Meriki 2013** | Cameroon | Cross-sectional | Adults attending TB and HIV care | 1,706 | 247 (14.5%) | Current smoker vs never smoker | TB-related AMR | MDR-TB | *Mycobacterium tuberculosis* | Crude | 1.15 (0.64–2.05) |
| **Mesfin 2018** | Ethiopia | Cross-sectional | TB patients | 226 | 43 (19.0%) | Ever smoker vs never smoker | TB-related AMR | MDR-TB | *Mycobacterium tuberculosis* | Adjusted | 3.52 (1.03–12.05) |
| **Min 2005** | Korea | Case-control | TB patients | 195 | 116 (59.5%) | Ever smoker vs never smoker | TB-related AMR | MDR-TB | *Mycobacterium tuberculosis* | Adjusted | 0.32 (0.14–0.77) |
| **Nguyen Thi Le Hang 2013** | Vietnam | Cross-sectional | TB patients | 546 | 323 (59.2%) | Ever smoker vs never smoker | TB-related AMR | Any drug-resistant TB | *Mycobacterium tuberculosis* | Adjusted | 1.87 (0.99–3.49) |
| **Olabiyi 2023** | Nigeria | Cross-sectional | TB patients | 1,203 | 122 (10.1%) | Ever smoker vs never smoker | TB-related AMR | MDR-TB | *Mycobacterium tuberculosis* | Crude | 1.33 (1.32–1.34) |
| **Otu 2014** | Nigeria | Cross-sectional | Pulmonary TB patients | 100 | Not reported | Current smoker vs never smoker | TB-related AMR | Any drug-resistant TB | *Mycobacterium tuberculosis* | Adjusted | 1.57 (0.47–5.27) |
| **Prakash 2016** | India | Cross-sectional | TB patients | 274 | 108 (39.4%) | Current smoker vs never smoker | TB-related AMR | MDR-TB | *Mycobacterium tuberculosis* | Crude | 4.46 (1.44–13.84) |
| **Queiroz 2002** | Brazil | Cohort | Patients with duodenal ulcer/erosive gastritis | 97 | 21 (21.6%) | Current smoker vs never smoker | Non-TB AMR | H. pylori treatment failure | *Helicobacter pylori* | Crude | 0.81 (0.20–3.29) |
| **Rifat 2014** | Bangladesh | Case-control | TB patients | 1,000 | 83 (8.3%) | Current smoker vs never smoker | TB-related AMR | MDR-TB | *Mycobacterium tuberculosis* | Adjusted | 0.04 (0.01–0.29) |
| **Romero Nieto 2021** | Spain | Cross-sectional | Patients with community-acquired acute pyelonephritis | 367 | 151 (41.1%) | Current smoker vs never smoker | Non-TB AMR | ESBL-producing/resistant Enterobacteriaceae/E. coli | *E. coli* | Adjusted | 4.85 (2.38–9.88) |
| **Ruddy 2005** | Russia | Cross-sectional | TB patients | 600 | Not reported | Current smoker vs never smoker | TB-related AMR | Isoniazid-resistant TB | *Mycobacterium tuberculosis* | Adjusted | 3.30 (1.20–9.20) |
| **Sahebi 2016** | Iran | Cross-sectional | HIV-negative smear-positive pulmonary TB patients | 280 | Not reported | Current smoker vs never smoker | TB-related AMR | Any drug-resistant TB | *Mycobacterium tuberculosis* | Adjusted | 1.16 (0.08–16.68) |
| **Salindri 2016** | Georgia | Cohort | MDR-TB patients | 267 | Not reported | Current smoker vs never smoker | TB-related AMR | Any drug-resistant TB | *Mycobacterium tuberculosis* | Adjusted | 4.56 (1.49–14.02) |
| **Sambas 2020** | Saudi Arabia | Cross-sectional | TB patients | 158 | 42 (26.6%) | Ever smoker vs never smoker | TB-related AMR | MDR-TB | *Mycobacterium tuberculosis* | Adjusted | 72.10 (2.41–2157.80) |
| **Sharma 2019** | India | Case-control | TB patients | 741 | 178 (24.0%) | Current smoker vs never smoker | TB-related AMR | MDR-TB | *Mycobacterium tuberculosis* | Adjusted | 2.56 (1.19–3.26) |
| **Sichen 2023** | China | Cross-sectional | TB patients | 395 | 96 (24.3%) | Ever smoker vs never smoker | TB-related AMR | MDR-TB | *Mycobacterium tuberculosis* | Crude | 1.00 (0.43–2.29) |
| **Sinha 2017** | India | Cross-sectional | Clinically suspected TB patients | 721 | 389 (54.0%) | Ever smoker vs never smoker | TB-related AMR | MDR-TB | *Mycobacterium tuberculosis* | Crude | 0.43 (0.21–0.87) |
| **Skrahina 2013** | Belarus | Cross-sectional | TB patients | 1,420 | 1,056 (74.4%) | Ever smoker vs never smoker | TB-related AMR | MDR-TB | *Mycobacterium tuberculosis* | Adjusted | 1.50 (1.10–2.00) |
| **Souba Diandé 2019** | Burkina Faso | Cross-sectional | TB patients | 1,250 | 323 (25.8%) | Ever smoker vs never smoker | TB-related AMR | Rifampicin-resistant TB | *Mycobacterium tuberculosis* | Crude | 1.04 (0.50–2.08) |
| **Wijayanto 2019** | Indonesia | Case-control | TB patients | 90 | 46 (51.1%) | Current smoker vs never smoker | TB-related AMR | MDR-TB | *Mycobacterium tuberculosis* | Crude | 0.71 (0.30–1.60) |
| **Zhang 2016** | China | Case-control | TB patients | 578 | 355 (61.4%) | Current smoker vs never smoker | TB-related AMR | MDR-TB | *Mycobacterium tuberculosis* | Adjusted | 2.07 (1.66–3.19) |
| **Zhu 2020** | China | Cross-sectional | TB patients | 1,831 | 552 (30.1%) | Current smoker vs never smoker | TB-related AMR | Any drug-resistant TB | *Mycobacterium tuberculosis* | Adjusted | 1.78 (1.17–2.28) |

Supplementary Table S2. AMR specimen/source and antimicrobial agents tested

| **Study** | **AMR outcome** | **Specimen / AMR ascertainment** | **Organism** | **Antibiotics / resistance markers** |
| --- | --- | --- | --- | --- |
| **Admassu 2023** | MDR-TB | Specimen not specified in extraction file; M. tuberculosis drug susceptibility testing reported | *Mycobacterium tuberculosis* | Isoniazid and rifampicin |
| **Ahmad 2016** | Fluoroquinolone-resistant TB | Sputum specimen; AMR assessed by culture/DST or molecular method as reported | *Mycobacterium tuberculosis* | Ofloxacin |
| **Al-Shahrani 2021** | MDR-TB and/or mono-resistance | Sputum specimen; AMR assessed by culture/DST or molecular method as reported | *Mycobacterium tuberculosis* | Isoniazid, Rifampicin, Pyrazinamide, Ethambutol, Streptomycin |
| **Ali 2019** | MDR-TB | Specimen not specified in extraction file; M. tuberculosis drug susceptibility testing reported | *Mycobacterium tuberculosis* | Isoniazid and rifampicin |
| **An 2020** | MDR-TB | Sputum specimen; AMR assessed by culture/DST or molecular method as reported | *Mycobacterium tuberculosis* | Rifampicin, isoniazid, streptomycin, ethambutol |
| **Balaji 2010** | MDR-TB | Mycobacterial culture isolate; drug susceptibility testing | *Mycobacterium tuberculosis* | Isoniazid, rifampicin, ethambutol, streptomycin, ciprofloxacin, ethionamide, capreomycin, cycloserine |
| **Dalton 2012** | XDR-TB | Sputum (culture) | *Mycobacterium tuberculosis* | Fluoroquinolones and second-line injectable agents: kanamycin, amikacin, capreomycin |
| **El Mahalli 2015** | Any DR-TB | Sputum (AFB smear); records review | *Mycobacterium tuberculosis* | First-line anti-TB drugs |
| **Elduma 2019** | MDR-TB | Specimen not specified in extraction file; M. tuberculosis drug susceptibility testing reported | *Mycobacterium tuberculosis* | Isoniazid and rifampicin/rifampin |
| **Fregona 2017** | MDR-TB and/or mono-resistance | Sputum specimen; AMR assessed by culture/DST or molecular method as reported | *Mycobacterium tuberculosis* | Isoniazid, Rifampicin, Pyrazinamide, Ethambutol, Streptomycin |
| **Ignatyeva 2015** | MDR-TB | Sputum specimen; AMR assessed by culture/DST or molecular method as reported | *Mycobacterium tuberculosis* | Isoniazid, rifampicin, ethambutol, streptomycin, pyrazinamide, ofloxacin, kanamycin, amikacin, capreomycin |
| **Isea-Peña 2013** | Levofloxacin-resistant Streptococcus pneumoniae | Blood cultures, cerebrospinal fluid, pericardial fluid | *Streptococcus pneumoniae* | Levofloxacin |
| **Jokinen 2017** | MRSA bacteremia | Blood cultures | *Staphylococcus aureus* | Methicillin/oxacillin and penicillin |
| **Junaid K 2020** | MDR-TB | Sputum specimen; AMR assessed by culture/DST or molecular method as reported | *Mycobacterium tuberculosis* | Rifampicin, Isoniazid |
| **Kadri 2022** | MDR-TB | Sputum specimen; AMR assessed by culture/DST or molecular method as reported | *Mycobacterium tuberculosis* | Rifampicin, Isoniazid |
| **Lee 2009** | MDR-TB | Sputum specimen; AMR assessed by culture/DST or molecular method as reported | *Mycobacterium tuberculosis* | Isoniazid, Rifampicin, Pyrazinamide, Ethambutol |
| **Lema 2016** | MDR-TB | Sputum specimen; AMR assessed by culture/DST or molecular method as reported | *Mycobacterium tuberculosis* | Isoniazid, Rifampicin |
| **Ma L.; Gao M 2022** | Any drug-resistant TB | Sputum specimen; AMR assessed by culture/DST or molecular method as reported | *Mycobacterium tuberculosis* | Isoniazid, Rifampicin, Pyrazinamide, Ethambutol, Rifapentine |
| **Marahatta 2010** | MDR-TB | Sputum specimen; AMR assessed by culture/DST or molecular method as reported | *Mycobacterium tuberculosis* | Rifampicin, Isoniazid |
| **Marahatta 2015** | MDR-TB | Sputum culture and drug susceptibility testing | *Mycobacterium tuberculosis* | Isoniazid and rifampicin |
| **Meriki 2013** | MDR-TB | Sputum specimen; AMR assessed by culture/DST or molecular method as reported | *Mycobacterium tuberculosis* | Isoniazid, rifampicin, ethambutol, streptomycin |
| **Mesfin 2018** | MDR-TB | Sputum specimen; AMR assessed by culture/DST or molecular method as reported | *Mycobacterium tuberculosis* | rifampicin, isoniazid, streptomycin, ethambutol and pyrazinamide |
| **Min 2005** | MDR-TB | Sputum specimen; AMR assessed by culture/DST or molecular method as reported | *Mycobacterium tuberculosis* | Isoniazid, Rifampicin, Pyrazinamide, Ethambutol, streptomycin, kanamycin, prothionamide, cycloserine, para-aminosalicylic acid, ofloxacin |
| **Nguyen Thi Le Hang 2013** | Any drug resistance | Sputum specimen; AMR assessed by culture/DST or molecular method as reported | *Mycobacterium tuberculosis* | Isoniazid, Rifampicin, Streptomycin |
| **Olabiyi 2023** | MDR-TB | Sputum specimen; AMR assessed by culture/DST or molecular method as reported | *Mycobacterium tuberculosis* | Rifampicin (rifampicin), Isoniazid (isoniazid), Ethambutol (ethambutol), Levofloxacin (LEV), and protionamide/Ethionamide (protionamide/ethionamide). |
| **Otu 2014** | Any DR-TB | Sputum specimen; AMR assessed by culture/DST or molecular method as reported | *Mycobacterium tuberculosis* | Isoniazid, Rifampicin, Streptomycin, Ethambutol |
| **Prakash 2016** | MDR-TB | Sputum specimen; AMR assessed by culture/DST or molecular method as reported | *Mycobacterium tuberculosis* | Isoniazid, rifampicin, ethambutol, streptomycin |
| **Queiroz 2002** | H. pylori treatment failure | Gastric biopsy | *Helicobacter pylori* | Clarithromycin and furazolidone |
| **Rifat 2014** | MDR-TB | Sputum specimen; AMR assessed by culture/DST or molecular method as reported | *Mycobacterium tuberculosis* | Isoniazid and rifampicin |
| **Romero Nieto 2021** | ESBL-producing E. coli | Urine or blood culture isolate | *Escherichia coli* | β-lactam antibiotics; ESBL phenotype |
| **Ruddy 2005** | Isoniazid resistance | Sputum specimen; AMR assessed by culture/DST or molecular method as reported | *Mycobacterium tuberculosis* | Isoniazid |
| **Sahebi 2016** | Any DR-TB | Sputum specimen; AMR assessed by culture/DST or molecular method as reported | *Mycobacterium tuberculosis* | Isoniazid, Rifampicin, Streptomycin, Ethambutol |
| **Salindri 2016** | Any DR-TB (excluding M/XDR and MDR-TB) | Sputum specimen; AMR assessed by culture/DST or molecular method as reported | *Mycobacterium tuberculosis* | First-line anti-TB drugs |
| **Sambas 2020** | MDR-TB | Sputum microscopy, Chest X-ray, GeneXpert PCR and Culture and drug susceptibility testing | *Mycobacterium tuberculosis* | Streptomycin, Isoniazid, Ethambutol, Rifampicin, Pyrazinamide |
| **Sharma 2019** | MDR-TB | Sputum culture and drug susceptibility testing | *Mycobacterium tuberculosis* | Isoniazid and rifampicin |
| **Sichen 2023** | MDR-TB | Sputum specimen; AMR assessed by culture/DST or molecular method as reported | *Mycobacterium tuberculosis* | Ciprofloxacin, Amikacin, Capreomycin, Propylthioisoniazid, Isoniazid Aminosalicylate Tablets, Moxifloxacin, Clarithromycin, Rifabutin, Ethambutol, Isoniazid, Rifampicin, Streptomycin, Levofloxacin, Ofloxacin, Linezolid, Gatifloxacin |
| **Sinha 2017** | MDR-TB | Sputum specimen; AMR assessed by culture/DST or molecular method as reported | *Mycobacterium tuberculosis* | Rifampicin, ethambutol: Ethambutol, streptomycin: Streptomycin, isoniazid: Isoniazid |
| **Skrahina 2013** | MDR-TB | Sputum specimen; AMR assessed by culture/DST or molecular method as reported | *Mycobacterium tuberculosis* | Rifampicin, ethambutol: Ethambutol, streptomycin: Streptomycin, isoniazid: Isoniazid, Ofloxacin, Kanamycin, Amikacin, Capreomycin |
| **Souba Diandé 2019** | Rifampicin resistance | Sputum specimen; AMR assessed by culture/DST or molecular method as reported | *Mycobacterium tuberculosis* | Rifampicin, isoniazid |
| **Welekidan 2020** | MDR-TB | Sputum culture and drug susceptibility testing | *Mycobacterium tuberculosis* | Isoniazid and rifampicin |
| **Wijayanto 2019** | MDR-TB | Sputum culture and drug susceptibility testing | *Mycobacterium tuberculosis* | Isoniazid and rifampicin |
| **Zhang 2016** | MDR-TB | Sputum specimen; AMR assessed by culture/DST or molecular method as reported | *Mycobacterium tuberculosis* | isoniazid, rifampicin/rifampin, streptomycin, ethambutol, kanamycin and ofloxacin |
| **Zhu 2020** | Any drug-resistant TB | Sputum culture and drug susceptibility testing | *Mycobacterium tuberculosis* | isoniazid, rifampicin, ethambutol, streptomycin, levofloxacin, kanamycin |

Supplementary Table S3. ROBINS-E risk-of-bias assessment

| **Study** | **D1 Confounding** | **D2 Exposure** | **D3 Selection** | **D4 Post-exp.** | **D5 Missing** | **D6 Outcome** | **D7 Reporting** | **Overall** |
| --- | --- | --- | --- | --- | --- | --- | --- | --- |
| **Admassu 2023** | Low | High | Some concerns | Not applicable | Low | Low | Low | High |
| **Ahmad 2016** | Low | Some concerns | Low | Not applicable | Low | Low | Low | Some concerns |
| **Al-Shahrani 2021** | High | Some concerns | Some concerns | Not applicable | Some concerns | Low | Low | High |
| **Ali 2019** | Low | Some concerns | Some concerns | Not applicable | Low | Low | Low | Some concerns |
| **An 2020** | High | Some concerns | High | Not applicable | High | Low | Low | High |
| **Balaji 2010** | High | Some concerns | Some concerns | Not applicable | Low | Some concerns | Low | High |
| **Dalton 2012** | Low | Low | Low | Not applicable | Low | Low | Low | Low |
| **El Mahalli 2015** | Low | Some concerns | Low | Not applicable | Low | Some concerns | Low | Some concerns |
| **Elduma 2019** | Low | High | Low | Not applicable | Low | Low | Low | High |
| **Welekidan 2020** | Low | Some concerns | Low | Not applicable | Low | Some concerns | Low | Some concerns |
| **Fregona 2017** | Low | Some concerns | Some concerns | Not applicable | Some concerns | Low | Low | Some concerns |
| **Ignatyeva 2015** | Low | Some concerns | Low | Not applicable | Low | Low | Low | Some concerns |
| **Isea-Peña 2013** | High | Some concerns | Low | Not applicable | Low | Some concerns | Low | High |
| **Jokinen 2017** | High | Some concerns | Some concerns | Not applicable | Low | Some concerns | Low | High |
| **Junaid K 2020** | High | Some concerns | Some concerns | Not applicable | Low | Low | Low | High |
| **Kadri 2022** | High | Some concerns | Low | Not applicable | Low | Low | Low | High |
| **Lee 2009** | Low | High | Some concerns | Not applicable | Low | Low | Low | High |
| **Lema 2016** | Some concerns | High | Some concerns | Not applicable | Some concerns | Low | Low | High |
| **Ma L.; Gao M 2022** | Low | Some concerns | Low | Not applicable | Low | Low | Low | Some concerns |
| **Marahatta 2010** | High | Some concerns | Some concerns | Not applicable | Low | Low | Low | High |
| **Marahatta 2015** | Low | Some concerns | Some concerns | Not applicable | Low | Some concerns | Low | Some concerns |
| **Meriki 2013** | High | Some concerns | Low | Not applicable | Low | Some concerns | Low | High |
| **Mesfin 2018** | Low | Some concerns | Low | Not applicable | Low | Low | Low | Some concerns |
| **Min 2005** | Low | Some concerns | Some concerns | Not applicable | Low | Low | Low | Some concerns |
| **Nguyen Thi Le Hang 2013** | Low | Some concerns | Low | Not applicable | Low | Low | Low | Some concerns |
| **Olabiyi 2023** | High | Some concerns | Low | Not applicable | Low | Low | Low | High |
| **Otu 2014** | Low | Some concerns | Low | Not applicable | Low | Some concerns | Low | Some concerns |
| **Prakash 2016** | High | Some concerns | Some concerns | Not applicable | Some concerns | Some concerns | Some concerns | High |
| **Queiroz 2002** | High | Some concerns | Low | Not applicable | Low | Low | Low | High |
| **Rifat 2014** | Low | Some concerns | Low | Not applicable | Low | Some concerns | Low | Some concerns |
| **Romero Nieto 2021** | Low | Some concerns | Low | Not applicable | Low | Some concerns | Low | Some concerns |
| **Ruddy 2005** | Low | Some concerns | Low | Not applicable | Low | Some concerns | Low | Some concerns |
| **Sahebi 2016** | Low | Some concerns | Some concerns | Not applicable | Low | Low | Low | Some concerns |
| **Salindri 2016** | Low | Some concerns | Some concerns | Not applicable | Low | Low | Low | Some concerns |
| **Sambas 2020** | Low | Some concerns | Some concerns | Not applicable | Low | Low | Low | Some concerns |
| **Sharma 2019** | Low | Some concerns | Low | Not applicable | Low | Some concerns | Low | Some concerns |
| **Sichen 2023** | High | Some concerns | Low | Not applicable | Low | Low | Low | High |
| **Sinha 2017** | High | Some concerns | Low | Not applicable | Low | Low | Low | High |
| **Skrahina 2013** | Low | Low | Low | Not applicable | Low | Low | Low | Low |
| **Souba Diandé 2019** | High | Some concerns | Low | Not applicable | Some concerns | Low | Low | High |
| **Wijayanto 2019** | High | Some concerns | Low | Not applicable | Low | Some concerns | Low | High |
| **Zhang 2016** | Low | Low | Low | Not applicable | Low | Low | Low | Low |
| **Zhu 2020** | Low | Some concerns | Low | Not applicable | Low | Some concerns | Low | Some concerns |

D1, bias due to confounding; D2, bias arising from measurement of the exposure; D3, bias in selection of participants into the study or analysis; D4, bias due to post-exposure interventions; D5, bias due to missing data; D6, bias arising from measurement of the outcome; D7, bias in selection of the reported result. Low risk indicates low risk of bias across all applicable domains. Some concerns indicates that at least one applicable domain raised some concerns, but no domain was judged at high risk. High risk indicates high risk of bias in at least one domain, or concerns across multiple domains that were considered sufficient to substantially reduce confidence in the result. Not applicable indicates that the domain was not relevant to the study. Overall judgements were derived from the domain-level assessments.

Supplementary figures

**Figure S1.** Sensitivity analysis restricted to adjusted estimates. Random-effects REML meta-analysis restricted to the 28 studies reporting adjusted estimates.

**Figure S2.** Sensitivity analysis excluding potentially influential studies. Random-effects REML meta-analysis excluding Sambas 2020, Olabiyi 2023, and Rifat 2014.

**Figure S3.** Funnel plot for small-study effects. Funnel plot for the primary meta-analysis. Egger test did not indicate statistical evidence of small-study effects (p=0.55).
